# Immunomodulation by extracorporeal ozone-based bactericide system in porcine *Pseudomonas aeruginosa* septic shock

**DOI:** 10.1038/s41598-025-07408-5

**Published:** 2025-06-25

**Authors:** Henrik Rundgren, Johan Sjöholm, Sanja Juric, Pellina Janson, Joline Kolerud, Robert Wallin, Paul Skorup, Miklos Lipcsey, Mattias Günther

**Affiliations:** 1 Sangair AB, Stockholm, Sweden; 2 RISE Research Institutes of Sweden, Stockholm, Sweden; 3 Karolinska Experimental Research and Imaging Centre (KERIC), Stockholm, Sweden; 4 SciEd Solutions, Stockholm, Sweden; 5 Department of Medical Sciences, Uppsala University, Uppsala, Sweden; 6 Department of Surgical Sciences, Uppsala University, Uppsala, Sweden; 7 Section for Experimental Traumatology, Department of Neuroscience, Karolinska Institutet, Biomedicum – 8B, 171 77 Stockholm, Sweden

**Keywords:** Porcine sepsis model, Ozone, *Pseudomonas aeruginosa*, Antibiotic resistance, Intensive care, Medical research, Experimental models of disease, Preclinical research, Translational research, Microbiology, Bacteria

## Abstract

Sepsis is associated with substantial mortality rates. Traditional treatment strategies often fail to address the underlying dysregulation in immune response, necessitating novel therapeutic approaches. Ozone (O_3_) is an inorganic molecule with no evident function in the body. We investigated the properties of ozone, using a system of extracorporeal ozone blood treatment in *Pseudomonas aeruginosa* septic shock. We hypothesized that extracorporeal ozonation would decrease bacteria in blood, have immunomodulating properties, and improve organ function. In this 4-h sepsis model swine were allocated to *P. aeruginosa* (PA-103, ATCC 29260, CCUG31589) infusion and ozone treatment (n = 7) or *P. aeruginosa* infusion and no ozone treatment (n = 6). Bacteria were infused in a peripheral vein. Mean (SD) duration of ozone treatment was 134 (67) min. A single pass through the system decreased viable *P. aeruginosa* by 53%, mean 2193 to 1023 colony forming units/mL, mean of differences -1170 (95% CI − 1689 to − 651, *P* < 0.0001). No difference in viable bacterial concentration was detected in peripheral venous blood between groups (*P* = 0.68). IL-1β, IL-4, IL-6, IL-8 and IFN-γ decreased by ozonation. Classical and alternative complement pathways were not affected. Blood hemoglobin, hematocrit and noradrenaline doses decreased in the treatment group. Breathing frequency and pulmonary peak airway pressure decreased in the ozone treatment group. Median survival in ozone treatment was 134 min and no treatment 159 min, with no statistical difference. Extracorporeal ozone blood treatment modulated the immune response in *P. aeruginosa* septic shock, which decreased mostly proinflammatory cytokines and was associated with indications of decreased vascular permeability and improved lung function and warrants further investigation for potential use in clinical settings.

## Introduction

Bacterial infections can escalate to sepsis and septic shock, both of which are associated with high mortality rates^1,2^. While antibiotics are essential for managing severe infections, the rise of global antibiotic resistance has become a significant concern, now recognized by the World Health Organization as one of the top ten global public health threats^3,4^. Sepsis is characterized by a dysregulated immune response which leads to organ dysfunction. Traditional treatment strategies often fail to address this underlying dysregulation, necessitating novel therapeutic approaches. Immunomodulatory therapy holds promise by restoring immune balance and mitigating excessive inflammation^5^. Ozone, also known as trioxygen, is an inorganic molecule with the chemical formula O₃. It is formed from dioxygen (O₂) through the action of ultraviolet (UV) light and electrical discharges in the Earth’s atmosphere^6^. While an ozone-like compound has been identified in atherosclerotic plaques, no definitive evidence supports a physiological role for ozone in the human body^7^. Nonetheless, experimental and clinical studies suggest potential medical applications^6,8^. Ozone generates reactive oxygen species (ROS), including superoxide (O₂⁻), hydroxyl radicals (OH), hydrogen peroxide (H₂O₂), nitric oxide (NO), and hypochlorous acid (HOCl). These ROS are also produced by granulocytes and macrophages during infections, where they may exert bactericidal effects by overwhelming bacterial redox defenses with oxidative stress^9^. In vitro, ozonated water has been shown to destroy bacterial biofilms^10^, and it is hypothesized that ozone could also target bacteria in the bloodstream. Additionally, ozone has been found to reduce the concentrations of viable bacteria in milk, demonstrating potential applications in the dairy industry^11^. It has also been shown to kill *Staphylococcus aureus*, *methicillin-resistant Staphylococcus aureus* (MRSA), and *Pseudomonas aeruginosa* in cell culture media^12^. Our research demonstrated that ozone reduced viable *E. coli* concentrations in human whole blood using an extracorporeal system, which was also found to be technically feasible and physiologically tolerable in a porcine septic shock model^13^. *P. aeruginosa* is one of the most common gram-negative bacteria associated with severe hospital-acquired infections, particularly in intensive care units^14,15^. To date, it is not known whether ozone kills *P. aeruginosa* in a clinical model of sepsis. Therefore, we investigated ozone in porcine septic shock by *P. aeruginosa*. We hypothesized that extracorporeal ozonation would decrease bacteria in blood, have immunomodulating properties, and improve organ function.

## Methods

**Fig. 1:**
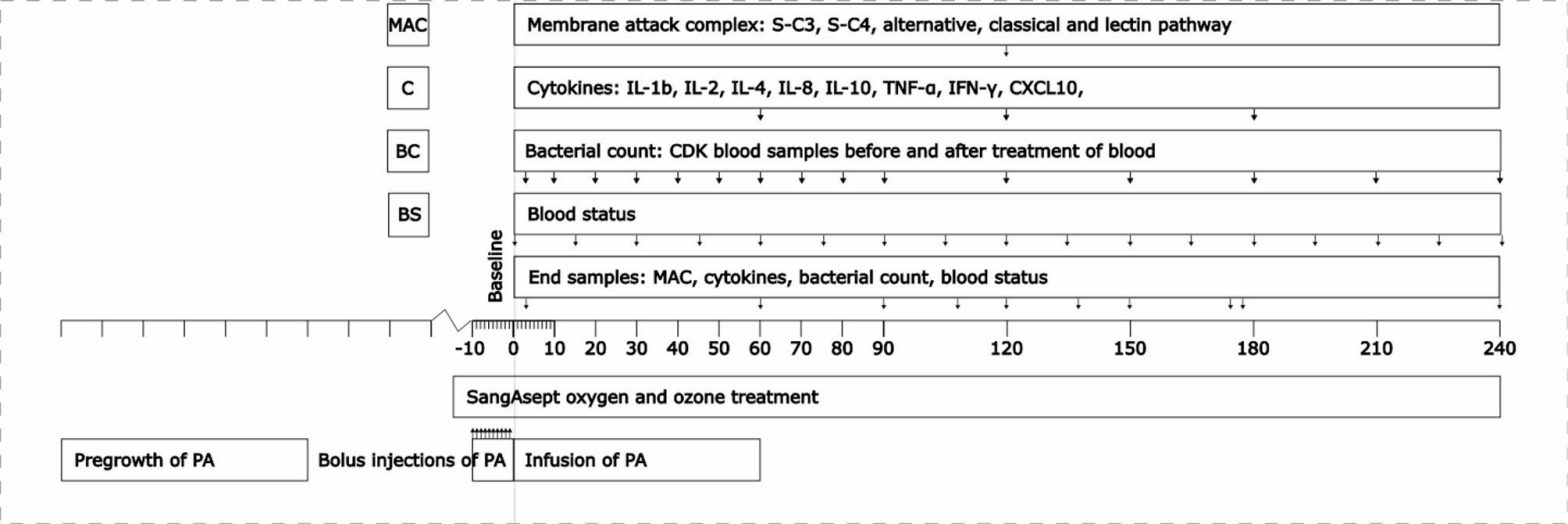
experimental setup of the swine sepsis model.

The animal experiments were approved by the Animal Ethics Committee in Linköping, Sweden (approval no: 3264, 12578-2020/17635-2020) and performed in agreement with the ARRIVE guidelines. The swine were handled in accordance with the Guide for the Care and Use of Laboratory Animals. The surgery was performed under general anesthesia and efforts were made to minimize suffering. Animals always had water ad libitum, and food until 12 h before the experiment. 13 specific-pathogen-free (SPF) Yorkshire/Swedish landrace crossbred swine (6 female, 7 male) with a mean weight of 42 (range 39–46) kg were provided from Company Johansson, Stockholm Region, Sweden. The experimental setup is described in Fig. 1.

### In vivo experiments

Pre-medication consisted of 150 mg tiletamine/zolazepam (Zoletil 100 Vet) and 6 mg medetomidine (Cepetor) after which anesthesia was induced with 0.2–0.3 mg alfentanil, 100 µg fentanyl (50 µg/mL) and 120–240 mg pentobarbital (60 mg/mL). Endotracheal intubation was performed with a Miller-type laryngoscope using a standard cuffed size 7 tube. Throughout the study, perioperative hypnosis was maintained with fentanyl 200–300 µg/hour and pentobarbital 800 mg/h. The animals were ventilated with a Dräger ventilator using pressure control with initial settings positive end-expiratory pressure (PEEP) 5, peak inspiratory pressure (PIP) 15 cm H_2_0, respiratory rate 15/min and FiO_2_ always remained at 21%. Settings were adjusted to maintain normoventilation (PaCO₂ 4.9–5.7 kPa). A 7.5 F, 110 cm pulmonary artery catheter (Edwards Lifescience) was inserted into the right internal jugular vein via cut-down for continuous monitoring of central venous pressure (CVP), cardiac output (CO), pulmonary artery pressure (PAP), mixed venous oxygen saturation (SvO₂), pulmonary arterial wedge pressure (PAWP), and core temperature (Vigilance CEDV monitor, Edwards Lifescience). Arterial blood gas parameters, including hemoglobin (Hb), methemoglobin (MetHb), pH, PaCO₂, PaO₂, Na⁺, K⁺, Ca^2^⁺, glucose, lactate, hematocrit (Hct), SaO₂, and base excess, were measured at baseline and at regular intervals throughout the experiment (ABL90 FLEX Plus). A 13.5 F, 15 cm double-lumen dialysis catheter (MedCOMP) was placed in the right femoral vein to facilitate ozone therapy. Continuous perioperative monitoring included electrocardiography and urine output. An initial fluid bolus of 500 mL Ringer’s acetate was administered at anesthesia induction to correct preoperative fluid imbalances, followed by a maintenance infusion rate of 3 mL/kg/h. Additional boluses of 100 mL were administered if mean arterial pressure (MAP) dropped below 35 mmHg. To simulate an intensive care environment, animals were managed according to a protocol aimed at maintaining vital parameters within normal physiological limits, consistent with prior studies^13^ and clinical best practices for sepsis in an intensive care unit. This included the use of noradrenaline and vasopressin for hemodynamic instability if required^1^. After preparation, animals were assigned to one of two groups: *P. aeruginosa* infusion with ozone treatment (n = 7) or *P. aeruginosa* infusion without ozone treatment (n = 6). Live *P. aeruginosa* was infused through a peripheral vein catheter for 1 h starting at baseline. To prevent clotting in the extracorporeal ozone system, 4000 IU of heparin was administered intravenously 15 min before ozone therapy began, followed by a continuous infusion of 33 IU/kg/h throughout the treatment period. This was also given in the group not receiving ozone treatment. At the end of the experiment, or at the time of circulatory collapse, the animals were euthanized with 40 mL pentobarbital sodium (Alfatal Vet 100 mg/mL).

### Extracorporeal blood ozonation

**Fig. 2:**
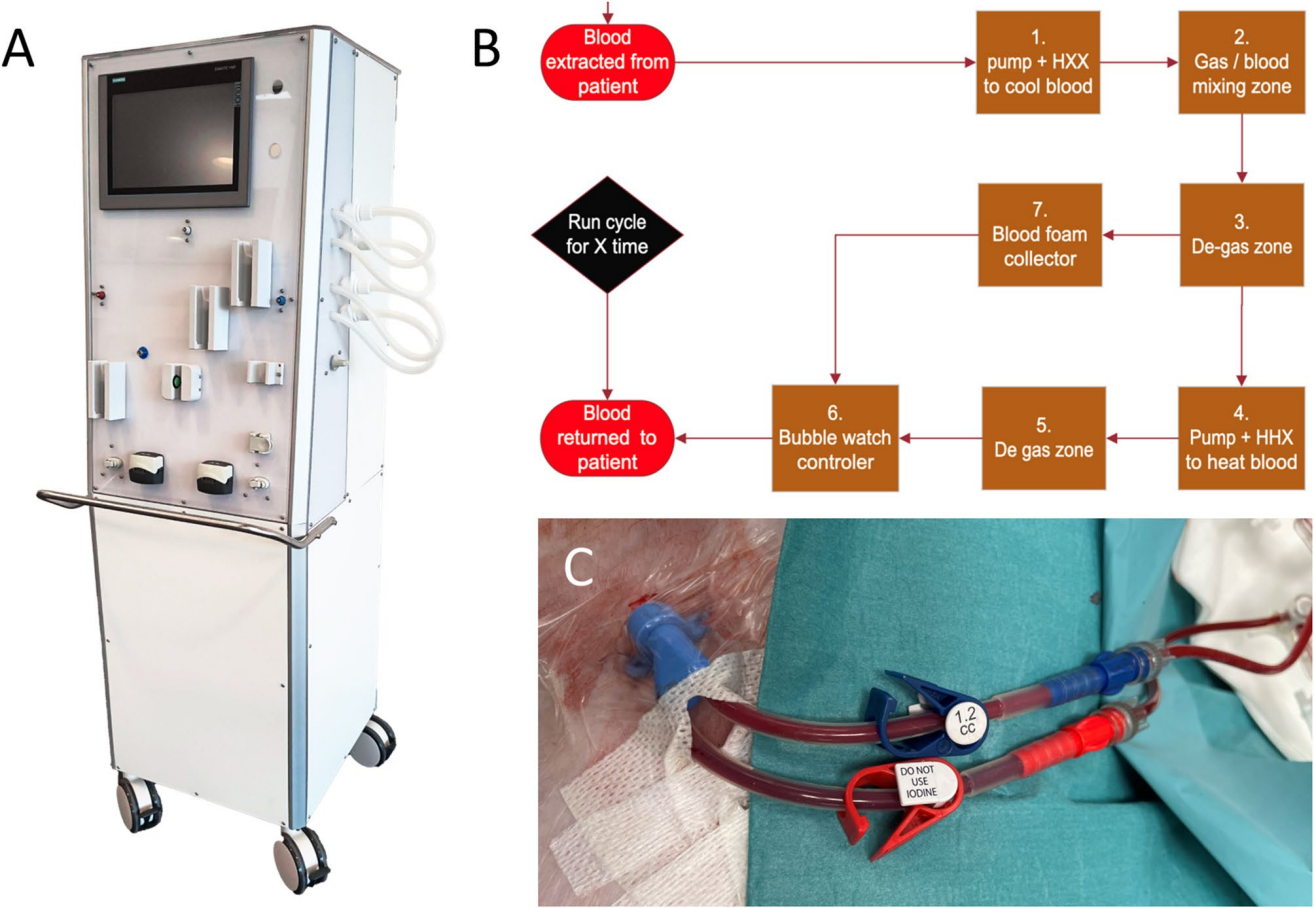
(**A**) Photo of the ozone prototype system (except connections). (**B**) Principal working mechanism of the ozone system. Venous blood is collected from the animal, decreased in temperature, mixed with ozone, reheated, and returned to the animal. (**C**) Photo of the central venous line (dialysis catheter) in the right femoral vein, showing dark red venous blood transported to the ozone system from the red connector, and light red, oxygenated blood returned from the oxygenator to the blue connector.

The SangAsept blood ozonation prototype (patent no. WO2016/043649) was used to treat blood with ozone. In this system, blood was drawn from the venous circulation of the swine at an average flow rate of 50 mL/min. The blood was first cooled to a median temperature of 4.1 °C, then exposed to a gaseous mixture of oxygen containing 3.1–4.7% ozone, as ozone solubility increases at lower temperatures^16^. The ozone concentration was maintained below 100 g/m^3^, with a gas flow rate of 15 mL/min. This cooling step optimized the ozonation process. After treatment, the blood was warmed to an average temperature of 29.6 °C before being returned to the swine. For in vitro studies, the treated blood was instead collected in a separate container (Fig. 2A,B).

### Organism

The bacteria strain, *P. aeruginosa* (PA-103, ATCC 29260, CCUG31589) was obtained from Uppsala University. The day before the experiment, bacteria from the master plate were streaked out on four Cystine Lactose Electrolyte Deficient agar plates (CLED) to form a lawn and incubated overnight at 37 °C. Bacteria were collected from the plates and used to inoculate 200 mL of Luria–Bertani broth that was grown under agitation at 37 °C for at least 90 min. Bacteria were pelleted by spinning the culture in 4 × 50 mL tubes at 2000xg (3549 RPM) for 5 min. The bacteria pellet was resuspended in 4 × 10 mL NaCl. The bacteria suspension was centrifuged again as previously described, the supernatant removed, the bacteria resuspended and diluted in NaCl to OD = 1.0. Thereafter, the bacteria suspension was diluted 1:2 in NaCl to an approximate concentration of 5 × 10^8^ colony-forming units (CFU)/mL. Injection and infusion of bacteria: two syringes for bolus injection and infusion were prepared with bacteria suspension with an approximate concentration of 5 × 10^8^ CFU/mL. The bolus was given as 10 injections of 1 mL in 10 min. The infusion was immediately thereafter given as a continuous infusion of 0.3 mL/kg/h for 1 h. Bolus injections and infusions were administered to the cephalic vein in the front leg and/or to the lateral auricular vein in the ear. T = 0 of the experiment was defined as the start of the infusion. At each sampling occasion, three samples of blood were withdrawn (0.5 mL in a 1 mL syringe). The samples were placed on wet ice and processed as swiftly as possible. Three baseline blood samples were withdrawn from the animal before connecting the SangAsept system to the animal. Three blood samples, 0.5 mL in 1 mL syringe, were withdrawn from the inlet to (i.e., before or upstream) and outlet (i.e., after or downstream) from SangAsept at each subsequent occasion. The samples were directly placed on wet ice and processed as swiftly as possible. The blood was transferred to sterile, cold 1.5 mL microcentrifuge tubes. 100 µl of each blood sample was transferred to new microcentrifuge tubes containing 900 µl of ice-cold sterile PBS and mixed (1:10 dilution). Thereafter 100 µl of 1:10 dilution was transferred to new microcentrifuge tubes containing 900 µl of ice-cold sterile PBS and mixed (1:100 dilution). 100 µl of each sample: undiluted, 1:10 and 1:100 sample was directly transferred to a cysteine lactose electrolyte deficient (CLED) plate and spread. The plates were transferred to an incubator, 37°C, for overnight culture. Scoring of CFU: the number of CFU on each plate was counted and noted. Bacterial quantification was performed by quantitative bacterial determination of blood on CLED agar plates (Becton Dickinsson). 0.1 mL blood was aliquoted on agar plates and cultured at 37 °C overnight and bacteria were quantified with viable count technique. Dialysis catheter blood samples of 0.1 mL were collected upstream and downstream from the extracorporeal ozone system before initiation of the treatment and after the 15 min of treatment had ended and aliquoted on CLED plates in triplicates for bacteria quantification (CFU per mL).

### Immunology

The Luminex assay, Porcine Premixed Multi-Analyte Kit (RnD Systems), was run according to the manufacturer’s instructions on a Bio-Rad Bio-Plex MAGPIX instrument. Briefly, the pig serum was diluted 1:2 with the calibrator diluent supplied with the kit. 50 µL of the diluted sample or standard mix was added per well of the 96-well sample plate. 50 µL of microparticle cocktail were then added and the plate was incubated on a horizontal orbital shaker for 2 h. The plate was then washed using a Bio-Rad Bio-Plex pro magnetic wash station, using the supplied wash buffer and the standard wash program. 50 µL of the Biotin-antibody cocktail were then added to the sample and standard wells and incubated on the orbital shaker for 1 h. The plate was then washed again, the same as before on the wash station, after which 50 µL of Streptavidin-PE solution was added and the plate incubated on the orbital shaker for 30 min. The plate was washed at the washing station and then the beads were resuspended and read on the Bio-Rad Bio-Plex MAGPIX instrument.

### Physiological calculations

Venous admixture (Qs/Qt) was calculated by the shunt equation (Berggren equation)^17^:

$${\text{Qs}}/{\text{Qt}} = ({\text{CcO}}_{2} - {\text{CaO}}_{2} )/({\text{CcO}}_{2} - {\text{CvO}}_{2} ),$$

where Qs/Qt = shunt fraction (shunt flow divided by total cardiac output), CcO_2_ = pulmonary end capillary O_2_ content, same as alveolar O_2_ content = SaO_2_ × Hb × 1.3. SaO_2_ was assumed to be 100% in the lungs. CaO_2_ = arterial O_2_ content (SaO_2_ × Hb × 1.3), CvO_2_ = mixed venous O_2_ content (SvO_2_ × Hb × 1.3). V′A/Q′ = alveolar minute ventilation/cardiac output. Units: CcO_2_/CaO_2_/CvO_2 _= mL/L, Hb = g/L, SaO_2_ = %.

### Statistical analyses

Statistical analyses were performed using GraphPad Prism version 10.3.0 for Windows (GraphPad Software, La Jolla, CA). The primary outcome was the concentration of viable *P. aeruginosa* in blood. *P* < 0.05 was considered statistically significant. Error bars represent the standard deviation. This study was a primary feasibility study which is why power calculation was not used. Temporal data sets were analyzed using a mixed-effects model (REML), fixed effects (type III) with Šídák's multiple comparisons test. We performed Shapiro–Wilk tests, confirming normality for all variables except MPAP, breathing frequency, and pO₂. However, residual analysis indicated that these approximated normality. For complement pathways, two-tailed, paired t-tests were used. For cumulative resuscitation fluids, an unpaired t-test was performed. Comparison of survival curves was done by Log-rank (Mantel-Cox) test. *P. aeruginosa* before and after SangAsept was not normally distributed and the non-parametric Wilcoxon matched-pairs signed rank test was used.

## Results

**Fig. 3:**
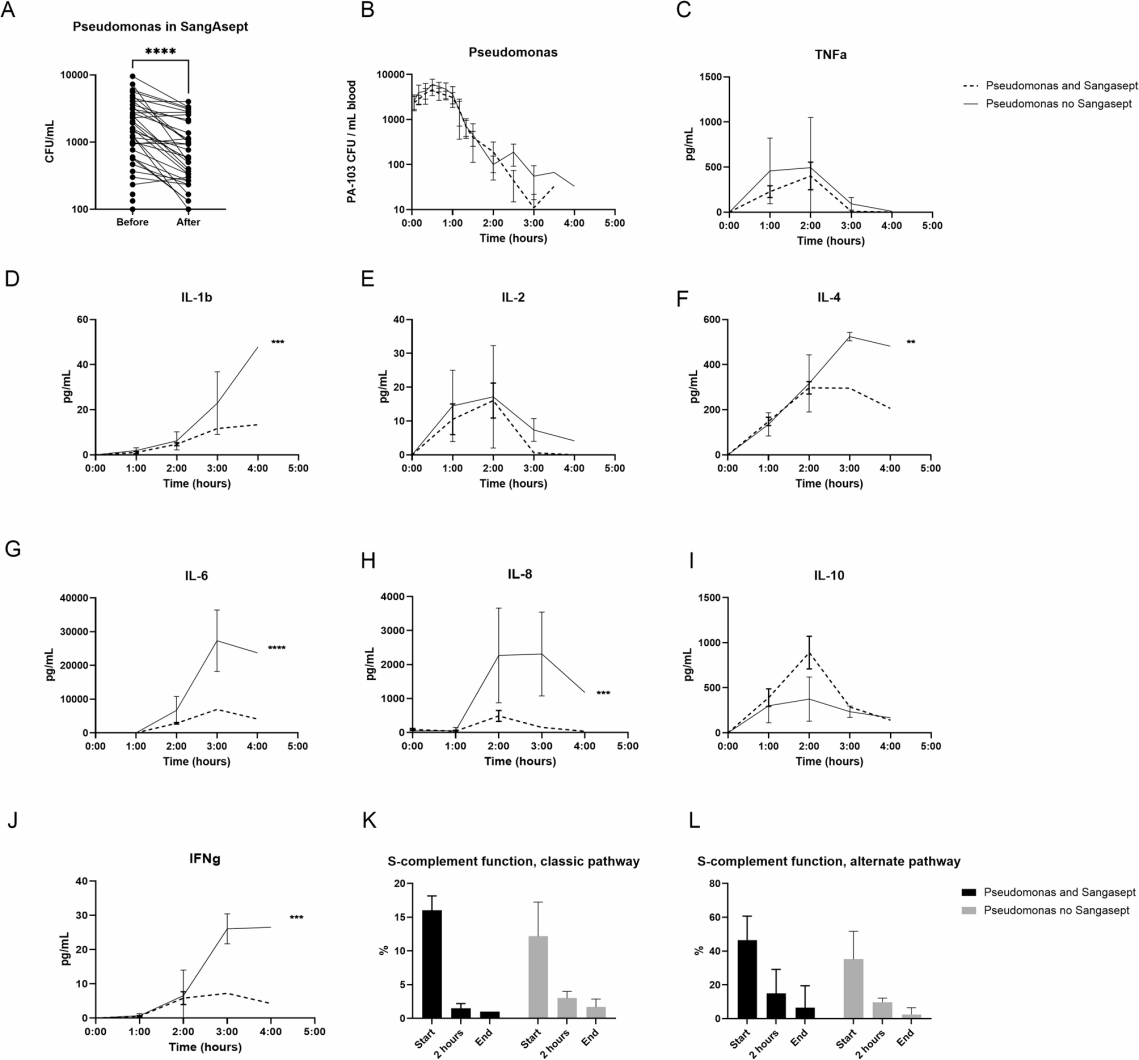
Bacterial and immunological response. (**A**) One pass of ozonation decreased *P. aeruginosa* by 53% before and after the system. (**B**) No difference in bacterial count in blood was detected. (**C**–**J**) IL-1β, IL-4, IL-6, IL-8, IFN-γ decreased by ozonation, and TNFa, IL-2, IL-10 were not affected. (**K**,**L**) Classical and alternative complement pathways were not affected. **P < 0.01, ***P < 0.005, ****P < 0.001.

The mean (SD) duration of ozone treatment was 134 (67) min. A single pass treatment decreased viable *P. aeruginosa* by 53%, mean 2193 to 1023 CFU/mL, mean of differences -1170 (95% CI -1689 to -651, *P* < 0.0001) (Fig. 3A). The bacterial bolus injection and infusion caused an increase in the concentration of viable bacteria during the first hour, after which a spontaneous decline was noted. No difference in bacterial concentration between groups was detected in peripheral venous blood (*P* = 0.6751) (Fig. 3B). TNFa did not differ (Fig. 3C). IL-1β decreased (*P* < 0.005) (Fig. 3D). IL-2 did not differ (Fig. 3E). IL-4 decreased (*P* < 0.01) (Fig. 3F). IL-6 decreased (*P* < 0.001) (Fig. 3G). IL-8 decreased (*P* < 0.005) (Fig. 3H). IL-10 did not differ (Fig. 3I). IFN-γ decreased (*P* < 0.005) (Fig. 3J). Complement function, classic pathway and alternative pathway, decreased at 2 h and end of experiments, compared to baseline. No differences were seen by ozone treatment (Fig. 3K,L).

**Fig. 4:**
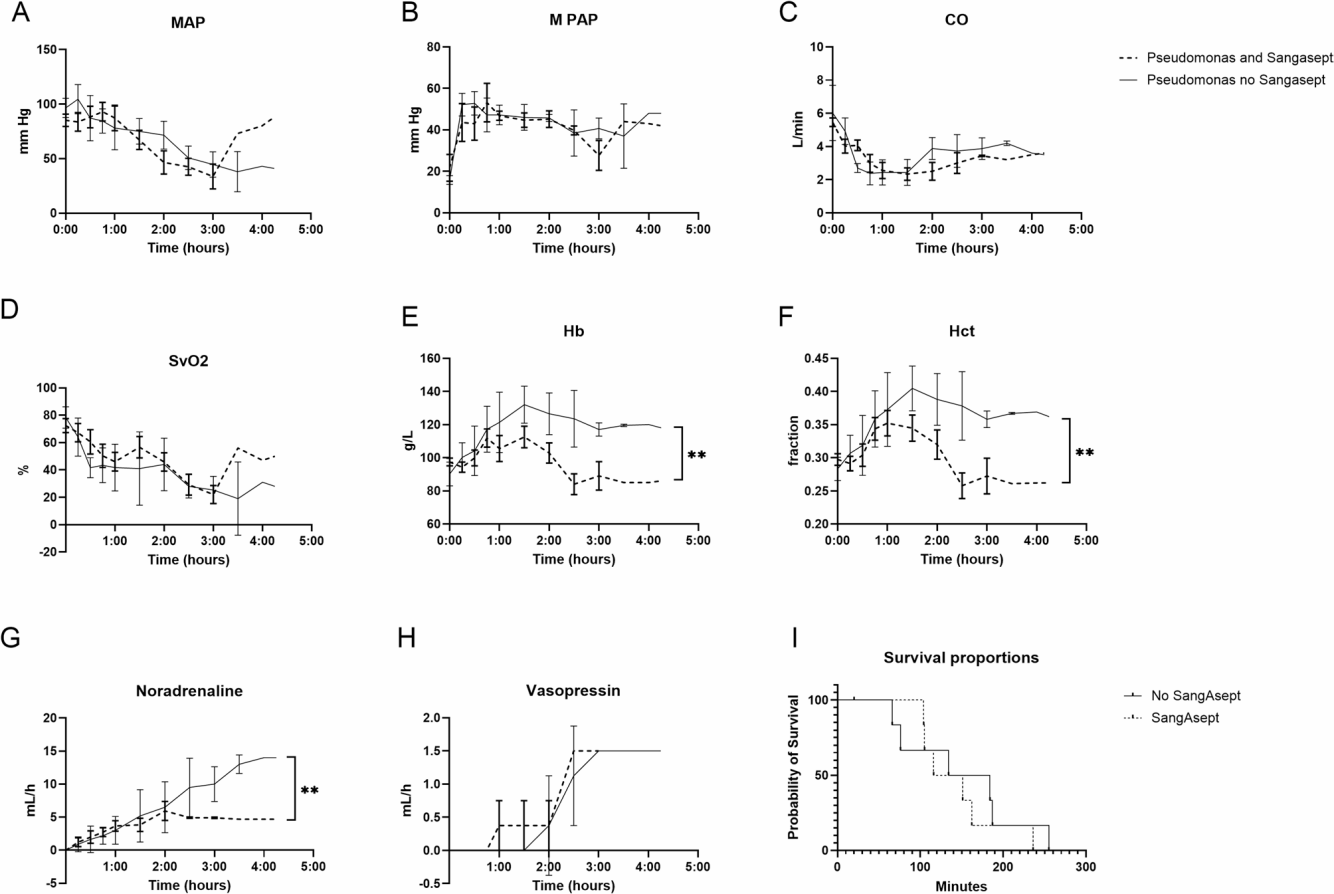
Circulatory response. (**A**–**D**) MAP, MPAP, CO and SvO_2_ did not differ between groups. (**E**,**F**) Hb and Hct decreased in the ozone treatment group. (**G**,**H**) Noradrenaline requirements decreased while vasopressin did not. (**I**) Median survival in ozone treatment was 133.5 min and no treatment was 159 min. No difference in survival was detected. **P < 0.01.

Circulatory effects from the sepsis induction and the ozone treatment were compared. MAP, MPAP, CO, and SvO_2_ did not differ between groups (Fig. 4A–D). Hb decreased in the ozone treatment group (*P* < 0.01) (Fig. 4E). Hct decreased (*P* < 0.01) (Fig. 4F). Noradrenalin doses to maintain blood pressure were lower in the ozone treatment group (*P* < 0.01) (Fig. 4G). Vasopressin doses did not differ between groups (Fig. 4H). Median survival in ozone treatment was 134 min and no treatment 159 min. No difference was detected between groups (*P* = 0.5843) (Fig. 4I). Mean (SD) cumulative resuscitation with Ringers´s acetate was 5767 (751) mL in the ozone treatment group and 8600 (2043) mL in the non-treatment group. No difference was detected between groups (*P* = 0.0654).

**Fig. 5:**
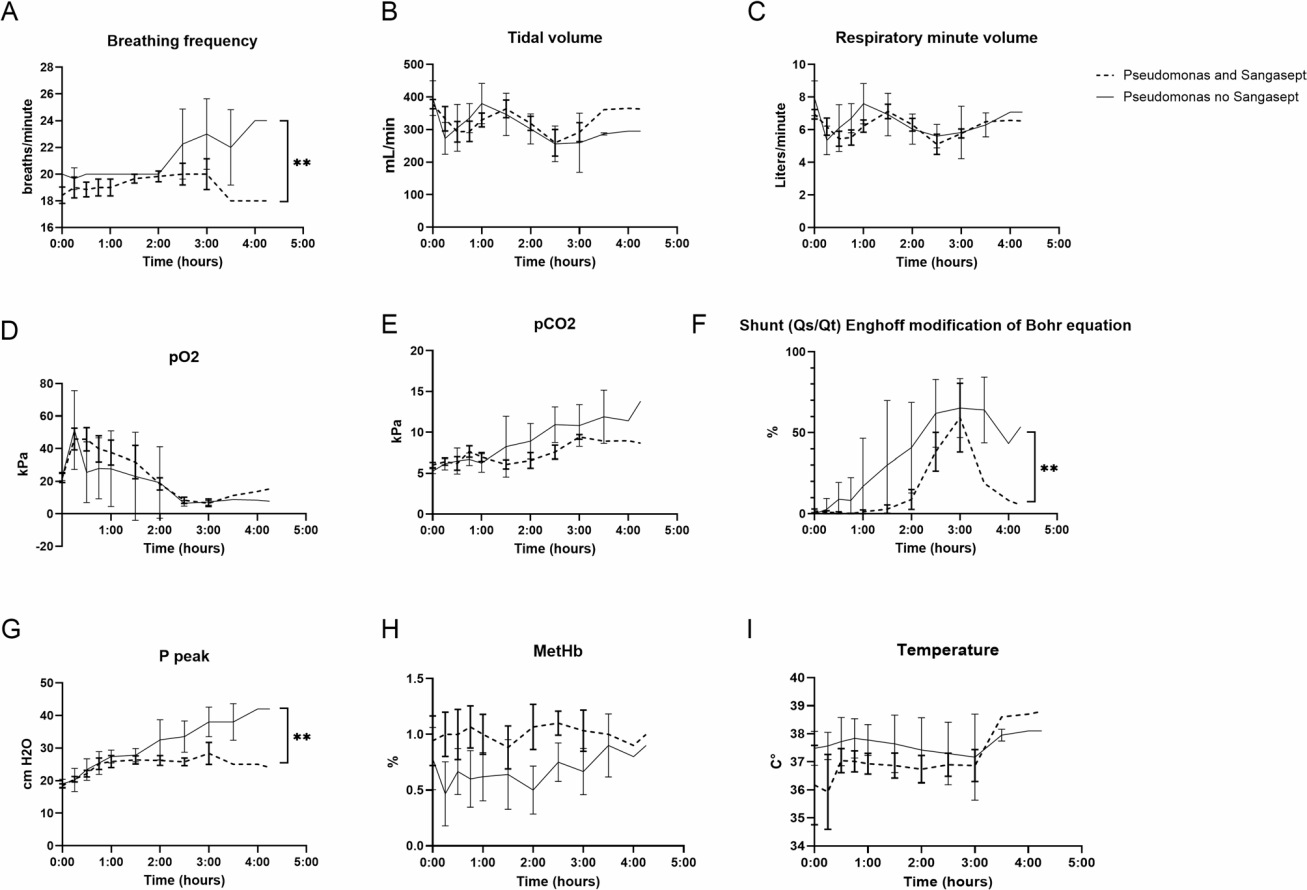
Respiratory response. (**A**–**G**) Breathing frequency, shunt and peak airway pressure decreased in the ozone treatment group. Tidal volume, respiratory minute volume, PaO_2_ and PaCO_2_ did not differ between groups. (**H**) Methemoglobin, a marker of oxidative stress, was not affected by ozone treatment. **P < 0.01.

Respiratory effects from the sepsis induction and the ozone treatment were compared. Breathing frequency decreased in the ozone treatment group (*P* < 0.01) (Fig. 5A). Tidal volume (Fig. 5B), respiratory minute volume (Fig. 5C), PaO_2_ (Fig. 5D) and PaCO_2_ (Fig. 5E) did not differ between groups. Pulmonary shunt decreased in the ozone treatment group (*P* < 0.01) (Fig. 5F). PIP decreased in the ozone treatment group (*P* < 0.01) (Fig. 5G). MetHb did not differ between groups (Fig. 5H). Core body temperature did not differ between groups (Fig. 5I).

## Discussion

In this study, we showed that extracorporeal ozonation modulated the immune response in *P. aeruginosa* sepsis. The modulation was cytokine-specific and did not affect complement activation. In addition, indications of decreased vascular permeability and improved lung function were detected.

We have earlier shown that extracorporeal ozonation of blood decreased the amount of viable *E. coli*^13^, but sensitivity to ozone may differ between strains. *P. aeruginosa* is a major cause of sepsis-derived mortality in hospitals^18^ and this study was conducted to investigate whether ozone would affect this strain. To simulate a clinical scenario where immediate and effective antibacterial treatment is critical, we employed a septic shock model using *P. aeruginosa* infusion. Median survival in this model was under 160 min, despite optimal resuscitation, reflecting the severity of the condition and the high mortality associated with such infections^19^. The bacterial infusion and study duration were informed by our prior experience and were designed to achieve measurable bacteremia ^13,20,21^. Ozone treatment was administered via a standard dialysis catheter inserted into the femoral vein. Treatment began 30 min prior to the bacterial infusion and continued for as long as conditions allowed. The system was compatible with an intensive care environment and did not induce any observable physiological adverse effects.

First, we investigated the effect on bacterial count. *P. aeruginosa* decreased with a rate of 53% by a single pass through the system, when measured in the blood flow before and after the chamber, which indicated that extracorporeal ozonation was able to decrease the number of viable bacteria. However, no difference was seen between groups when measuring bacteria in blood taken from a peripheral vein, i.e. blood that had been circulating in the swine, together with fluids, medical infusions and the bacterial infusion. To produce sufficient levels of bacteria in blood for accurate subsequent quantification in agar plates, a high infusion rate was chosen. This produced supra-normal levels of bacteria in the blood. It is possible that these levels surpassed the capacity of the ozonation system, and that lower levels would be manageable by the system. Moreover, while the magnitude of bacteremia may correlate with the severity of disease, other factors play more important roles in determining the patient’s outcome. Most episodes of clinically significant bacteremia are characterized by low numbers of bacteria per milliliter of blood^22^. It is possible that survival benefits would be detected in a less severe sepsis model, with lower levels of circulating bacteria, which should be the focus of future research.

Next, we quantified levels of circulating cytokines. Cytokines are a diverse group of small proteins, typically under 40 kDa, that include interleukins, chemokines, interferons, tumor necrosis factors, and growth factors. These molecules are primarily synthesized and secreted by immune cells. During an infection, the cytokine network is activated, consisting of both pro-inflammatory and anti-inflammatory cytokines. The interplay between these opposing regulatory pathways ultimately dictates the overall inflammatory response within the cytokine network^23^. We detected that IL-1β, IL-4, IL6, IL-8 and IFN-γ decreased by ozonation. IL-1β , IL6, IL-8 and IFN-γ are mostly proinflammatory while IL-4 is anti-inflammatory^24^. The effect was consistent during the observation period and was specific to cytokine levels; we also quantified levels of complement factors and found no effect by ozonation. Ozon has earlier been suggested to modulate IL-6 and IL-10^25^ and may stimulate the function of the peripheral blood cells^26^. The exact mechanism and relation to the outcome should be the focus of future investigations.

Then, circulatory effects were investigated. MAP decreased and MPAP increased because of sepsis^13^, which also caused CO and SvO_2_ to decrease. Interestingly, Hb and Hct decreased by ozonation, although the total amount of resuscitation with crystalloids did not differ between groups. It is possible that the decreased Hb and Hct were consequences of decreased vascular permeability—which retains water in the bloodstream and therefore decreases the relative concentration of Hb and subsequently Hct. A highly selective endothelial barrier is essential to maintain tissue fluid homeostasis and to support normal organ function^27^. In response to cytokines produced by immune cells, the endothelium expresses adhesion molecules and produces vasoactive compounds, inflammatory cytokines, and chemoattractants, which can lead to increased capillary permeability^23^. A main feature of the endothelium in sepsis is increased permeability or loss of barrier function, resulting in a shift of circulating elements and tissue edema^28^. Thus, it is possible that the decreased cytokine concentration in the blood caused a clinically measurable decreased vascular permeability, which should be confirmed in future investigations.

Then, respiratory effects were investigated. The ozonation group had decreased breathing frequency and peak airway pressures required to maintain respiratory minute volumes, and equal PaO_2_ and PaCO_2_. The lung is the earliest and most susceptible target organ in multiple organ dysfunction caused by sepsis, and up to 50% of sepsis patients experience acute lung injury (ALI). Patients with sepsis-induced ALI have weakened gas exchange function due to lung inflammation and tissue damage and pathological processes include pulmonary vascular endothelial damage, reduced alveolar surface tension, inflammatory factor release and pulmonary interstitial fibrosis^29^. Pulmonary shunt was then calculated. It decreased in the ozonation group, thus improving lung function. Pulmonary shunt refers to the passage of venous blood into the arterial blood system by passing the alveoli-blood gas exchange. Pulmonary shunting is a well-defined drop in the physiologic coupling of lung ventilation and lung perfusion in ALI^30^. Whether this also is a consequence of endothelial improved function remains to be elucidated.

Methemoglobin levels did not increase, consistent with findings from our previous investigation^13^. Methemoglobin is an oxidized form of hemoglobin (Hb), typically maintained at less than 1% of total hemoglobin under normal conditions. Clinically significant methemoglobinemia can arise from exposure to oxidizing agents, including certain medications^31^. The oxidative stress induced by ozone and its bactericidal effects must be balanced against potential adverse effects on blood by ensuring ozone doses remain within the neutralizing capacity of the blood’s antioxidative defenses^6,32^. In this study, the ozone dose may not have caused excessive oxidative damage; however, future studies should evaluate oxidative stress comprehensively, including markers of DNA damage and lipid peroxidation^33–35^.

Several limitations warrant discussion. First, the study lacked a control group without ozone treatment, as the primary objective was to evaluate the feasibility of ozone therapy in sepsis. Second, while swine are a well-accepted model for sepsis due to their similarities to humans in immune response and organ function^36,37^ and comparable anatomy and physiology^38^, the clearance of *P. aeruginosa* may be more efficient in swine than in humans^36^. This difference could affect the generalizability of the findings; nonetheless, swine remains a valid model for sepsis research^39^. Third, the study did not include animals that were not infused with *P. aeruginosa*. This decision was based on the study’s focus on evaluating the feasibility of ozone treatment in sepsis^20^, and the inclusion of such controls would not have impacted hypothesis testing in this feasibility study. Moreover, it aligns with the 3Rs principle of ethical animal research, specifically the principle of reduction. Fourth, while our prior experience with sepsis models informed the study design^13^, the 4-h observation period was likely too short to detect significant improvements in sepsis parameters. Future studies should consider longer observation times. Fifth, the study did not directly measure endotoxin or other PAMPs, which play a key role in immune dysregulation during Gram-negative sepsis. While our findings suggest that immunomodulation was not solely due to bacterial killing, future studies should quantify endotoxin levels to better elucidate the mechanism of ozone’s effects. Sixth, in this proof-of-concept study, the extracorporeal ozone treatment was administered at a flow rate of 50 mL/min, treating approximately 2 L of blood over the 4-h experimental period. While this setup allowed for an initial evaluation of the method’s feasibility, we recognize that a higher flow rate or extended treatment duration may be necessary for meaningful clinical effects. The relatively low volume of treated blood could explain the lack of significant reduction in systemic bacterial load despite the localized bactericidal effect observed in the extracorporeal circuit. Future studies should explore optimized flow rates, prolonged treatment durations, or intermittent cycles of ozonation to enhance systemic bacterial clearance and maximize therapeutic potential. Seventh, the discrepancy between bacterial reduction in the extracorporeal circuit and the unchanged systemic bacterial load suggests that while ozone effectively kills bacteria within the treatment loop, continuous bacterial influx from circulation counteracts this effect. This highlights a key limitation of the current setup, where the flow rate and duration of treatment may not be sufficient to achieve a sustained reduction in systemic bacterial burden. Additionally, while cooling may contribute to the observed effects, its exact role remains unclear. Future studies should focus on optimizing flow rates, extending treatment duration, and isolating the specific contributions of ozone exposure and temperature changes to fully understand their impact on bacterial clearance and immune modulation. Eighth, ozone treatment was initiated near the onset of sepsis for methodological reasons, which may limit the clinical applicability of the findings. Ninth, the study lacked a control circuit with identical flow, tubing, cooling, and rewarming but without ozone. Although the materials and setup closely resemble those used in clinical dialysis systems—which are not known to affect cytokine levels, future studies should include an ozone-free circuit to isolate the specific contribution of ozone from possible confounding physical factors. Finally, the interpretation of decreased hemoglobin and hematocrit as markers of reduced vascular permeability is hypothetical, as no direct measurements (e.g., Evans blue or wet/dry ratio) were performed. While the groups received similar fluid volumes, alternative explanations such as hemodilution or sampling variability cannot be fully excluded and should be addressed in future studies.

While a 53% reduction does not meet the bactericidal threshold of ≥ 3 log₁₀, it may still lower the bacterial burden faced by the immune system. This partial clearance, though modest, coincided with measurable physiological effects such as reduced cytokines and improved pulmonary parameters. Thus, even sub-bactericidal reduction could be clinically relevant, especially in systems with higher flow or prolonged use.

The ozonation system comprises a new method of modifying the immune response in sepsis. Immunomodulatory therapy in managing sepsis, a condition marked by a dysregulated immune response to infection, is likely pivotal for improving mortality^5^. The endothelium undergoes dramatic changes during sepsis and remains one of the most compelling targets for therapeutic development. However, the modalities recommended for the management of sepsis do little to protect the endothelium or restore endothelial function, leaving much room for future drug development^23,40^. Integrating such therapies into sepsis management protocols, particularly for patients with severe or refractory disease, holds the potential to reduce mortality. The current prototype has limited blood flow capacity (~ 50 mL/min), but the design is inherently scalable. Higher capacity systems are under development, which could allow full extracorporeal blood processing, similar to renal replacement therapy platforms used in ICUs.

## Conclusion

Extracorporeal ozone blood treatment modulated the immune response in *P. aeruginosa* septic shock, which decreased cytokines and was associated with indications of decreased vascular permeability and improved lung function and warrants further investigation for potential use in clinical settings.

## Data Availability

The datasets used and/or analyzed during the current study are available from the corresponding author on reasonable request.
